# Visuospatial Working Memory Tasks May Not Reduce the Intensity or Distress of Intrusive Memories

**DOI:** 10.3389/fpsyt.2022.769957

**Published:** 2022-02-03

**Authors:** Amalia Badawi, Zachary Steel, David Berle

**Affiliations:** ^1^Graduate School of Health, University of Technology, Sydney, NSW, Australia; ^2^School of Psychiatry, University of New South Wales, Sydney, NSW, Australia; ^3^St John of God Health Care, Richmond Hospital, Sydney, NSW, Australia; ^4^Black Dog Institute, Randwick, NSW, Australia

**Keywords:** intrusive memories, intensity, distress, visuospatial cognitive interventions, trauma

## Abstract

Cognitive interventions involving visuospatial tasks, such as the game “Tetris” have demonstrated efficacy in reducing the frequency of intrusive memories. However, it remains unclear whether these tasks also reduce the perceived intensity and distress of these memories. We investigated whether either of two visuospatial tasks: a Tetris intervention or Digital Corsi task, following the viewing of an analog trauma (film) resulted in decreased intensity and distress for intrusive memories over the following week, when compared to a control condition. Participants (*n* = 110) were randomly assigned to task conditions after viewing the film. Linear mixed models indicated no between-group differences for reductions in intensity or distress over the course of the week. These findings highlight an important boundary to the benefits of such visuospatial tasks, in that while they may be associated with reductions in intrusion memory frequency, individuals may nonetheless continue to experience distress when intrusions do occur.

## Introduction

Intrusive memories are a characteristic feature of acute stress disorder and posttraumatic stress disorder (PTSD). They are involuntary, repeated, and distressing recollections following exposure to a trauma event that most commonly take the form of visual mental images (1, 2). Intrusive symptoms are predictive of further PTSD symptomatology. Network analysis has demonstrated a predictive relationship between intrusive memories and other PTSD symptoms in the acute phase following trauma event exposure (3). Intrusive symptoms measured 10-months after direct exposure to a terrorist attack have been shown to strongly predict posttraumatic symptom clusters 2-years after the event (4). These findings suggest an important role for targeted interventions that decrease the influence of intrusive memories on pathology following exposure to a traumatic event.

Cognitive interventions involving a taxing visuospatial task (henceforth “visuospatial” tasks), such as the Tetris intervention and complex pattern tapping, have demonstrated efficacy for reducing the trajectory of intrusive memory frequency following exposure to an analog traumatic event, when administered in the memory consolidation phase (5–8). Memory consolidation, first referred to over a century ago by Muller and Pilzecker (9), is the stabilization of memories involving transition of memories from short- to long-term storage via protein synthesis (10). The consolidation process is said to occur within 6-h of exposure to an event (11). Given the outcomes of earlier studies showing decreased intrusive memory frequency after engagement in a visuospatial task during the memory consolidation window (5–8), it is possible that these same tasks will also influence the intensity and distress levels associated with the intrusive memories.

The “visuospatial hypothesis” [(6), p. 759] proposes a mechanism by which visuospatial tasks influence memory consolidation: It is thought that the consolidation of sensory-based information requires working memory resources which are disrupted by visuospatial tasks that compete for this limited pool of working memory resources. This disruption to the consolidation of sensory-based trauma-related memories is thought to in turn result in fewer intrusive memories. An important consideration with respect to the disruption of memory consolidation is that working memory resources need to be taxed in a similar sensory domain to that of the memory itself. For example, Tetris game play has been correlated with taxing visuospatial, but not other forms of working memory (12). Support for the premise of finite working memory resources is also indicated by studies showing that greater benefit for emotionality of negative memories was derived when tasks required only mild or moderate demand, as compared to when working memory was highly taxed (13).

As trauma processing appears to be hampered by avoidance strategies associated with the distress of recalling trauma memories (14), an increased understanding of emotionality for intrusive memories may help to inform treatment planning. This is particularly relevant in light of the cognitive model of PTSD, which posits that avoidance strategies (rumination, dissociation) in relation to intrusive memories are maintaining factors in PTSD (14, 15). In a study investigating responses to intrusive memories in trauma-exposed people, increased levels of anger and shame were indicated for participants with PTSD when compared to participants without this diagnosis (16). Such findings suggest that emotional responding to intrusive memories may be a crucial factor in the maintenance of psychopathology following trauma exposure. Therefore, directly assessing the perceived intensity and distress associated with intrusive memories may also serve as an important indicator of trauma recovery.

Promising findings in relation to the influence of visual and spatial tasks on reduced emotionality for intrusive memories have been indicated in studies that used either eye-movements or spatial tasks to assess emotional outcomes in relation to intrusive memories. Andrade et al. (17) asked participants to generate mental imagery after viewing photographs of distressing events such as war and death. They reported that the eye movement and complex tapping conditions were associated with decreased emotionality and vividness when compared to controls (Experiment 4). Decreased emotionality and vividness have also been reported for negatively valenced autobiographical memories in non-clinical (18, 19) and clinical PTSD (20) samples following engagement in eye-movement tasks.

Although few studies have investigated the influence of visuospatial tasks on the intensity of intrusive memories, Englehard et al.'s (13) study showing reduced emotional intensity for mental imagery following recall of negative events when followed by arithmetic tasks provides support for the investigation of intensity as a distinct characteristic of intrusive memories. In contrast, a recent study by Meyer et al. (21) did not find evidence for decreased levels of intrusion related distress following administration of a trauma film. Despite these inconsistencies in the literature, the potential for decreasing the intensity and distress of intrusive memories among trauma survivors means that these approaches warrant additional investigation.

Previous studies that utilized visuospatial tasks during memory consolidation have tended to assess levels of distress for the trauma event [e.g., (7, 22, 23)], rather than the intensity or distress associated with the intrusive memories themselves. In this paper, we investigated the trajectory of intensity and distress associated with intrusive memories following engagement in a visuospatial task after exposure to an analog trauma using a trauma film paradigm. We hypothesized that participants who received a visuospatial task (either Tetris or Digital Corsi) would report lower intensity and distress levels for intrusive memories of the film over the week as compared to participants in the control condition.

This extends our pre-registered program of research (https://osf.io/snp8g) beyond our previous replication paper (5), which focused on the frequency of intrusive memories in an effort to affirm the findings of the landmark study by Holmes et al.'s (8) showing the influence of the Tetris intervention on reducing the frequency of intrusive memories for an analog trauma. Investigation of intrusive memory intensity and distress was beyond the scope of that paper.

## Method

### Participants

English speaking participants aged 18 and over from the community were recruited using online advertisements and posters. For ethical purposes, people who reported recent involvement in a motor vehicle accident, current suicidal ideation or thoughts of self-harm, or active traumatic stress symptoms were excluded from participating. Ethical approval was obtained from the Human Research Ethics Committee at the University of Technology Sydney (ETH17-1455) and all participants provided informed consent. Participants who completed all components of the study received a $30 e-gift voucher.

### Power

Medium to large between-group effect sizes have been reported in previous studies that investigated the influence of the Tetris intervention on intrusion frequency following exposure to analog trauma (e.g., *d* = 0.91, 7; *d* = 0.62 and 0.70, 8). In the present study, 26 participants were needed per group condition in order to detect a large between-group effect size with a two-tailed α of 0.05 and 80% power, though we oversampled to allow for attrition and smaller than expected effect sizes.

### Tasks and Materials

Data were collected within the context of a broader study investigating intrusive memories (5). The interested reader is referred to Badawi et al. (5) for a comprehensive overview of study methodology. Study protocols, materials and de-identified data are accessible at https://doi.org/10.17605/OSF.IO/VUTCW.

#### Self-Report Questionnaires

##### Demographics

Participant age, gender, main occupation/field of study, and first language were obtained using a web-based questionnaire. The following well-validated self-report questionnaires were administered, with reliability levels in the current sample as indicated below. Depression symptoms were assessed using the Patient Health Questionnaire-9 (24) (PHQ-9; Cronbach's α = 0.84). The PROMIS Short Form v1.0—Anxiety 7a (25) was used to assess anxiety disorder symptoms (Cronbach's α = 0.91). Re-experiencing symptoms were assessed on Day 8 using the Impact of Events Scale—Revised (26) (IES-R; Cronbach's α = 0.88). Participants also completed measures for rumination, dissociation and intolerance of uncertainty.

#### Trauma Film Paradigm

The Trauma Film Paradigm has been used extensively in previous research [e.g., (7, 8, 27)] and was used as an analog trauma condition designed to elicit intrusive memories. The film consisted of 11 different video clips involving themes of death and injury, and ran for 10.5 min.

#### Visual Analog Scales

Online visual analog scales (VAS; 0–100) were used to assess levels of attention to the Trauma Film Paradigm; mood ratings (sad, hopeless, fearful, depressed, horrified) before and after viewing the Trauma Film Paradigm; attention given to the film; emotionality for intrusive memories following the 4-h break; task enjoyment; and diary accuracy.

#### Film Reminder Cue

Consistent with previous studies [e.g., (6, 8)], prior to the experimental task, a static visual image from each clip that was part of the film paradigm was shown for 3-s using a PowerPoint slideshow.

#### Cognitive Intervention Including Tetris

The three-part cognitive intervention including Tetris involved mental rotation practice of blocks in the game, a reminder cue for the trauma event, and Tetris gameplay where the player has visibility of the upcoming blocks and imagines rotating them for optimal placement. The computer game Tetris (UTS Research Version) (28) involves players rotating descending different shaped and colored blocks in order to make horizontal lines, which disappear and result in the player increasing their score. This version allowed for 12-min of continuous gameplay. Participants were able to view the three upcoming blocks and participants were asked to mentally imagine rotating the blocks to successfully complete the maximum number of horizontal lines.

#### Cognitive Intervention Including Digital Corsi

A 12-min adaptation of the original Corsi tapping task (29) was administered on a laptop computer. The blocks were nine digital blue squares that flashed yellow for 800 ms in pre-set, gradually increasing, sequences. Participants were required to view each sequence and to then click on the boxes in the corresponding order for 8-min and in backward order for 4-min. The cognitive intervention including Digital Corsi is henceforth referred to as “D-Corsi”.

#### Intensity and Distress Measures: Diary for Intrusive Memory Recording

Intrusive memory characteristics were assessed using the Metricwire app (Version 4.1.2) (30) on participants' IOS or Android phone device. On Day 0, a single diary entry was made 8:30 pm. On Days 1 to 7, surveys were made available three times per day (8:30 am, 2:30 pm, 8:30 pm), allowing participants to record data for frequency, intensity, and distress at 22 time points. The app was programmed so that participants were sent a notification at the time of the surveys being available and also 30-min later if no response had been recorded. The survey commenced with a brief description of intrusive memories and participants recorded information pertaining to frequency, intensity, and upsetting. *Intensity and distress, as indicated by levels of “upsetting”, were measured using online visual analog scales (VAS), which allowed participants to drag the slider along a continuum anchored from 0 (not at all) to 10 (extremely) to indicate their endorsement level*.

The use of this ecological momentary assessment method meant participants could repeatedly capture data while in their natural environment, thereby supporting ecological validity of the information gathered (16, 31). This method is consistent with previous studies that have assessed intrusive memory frequency, following trauma exposure, over the course of several days [e.g., (6–8, 16, 23)].

### Procedure

#### Day 0: Morning Session

After providing informed consent, participants completed the self-report measures, VAS (sad, hopeless, fearful, horrified, depressed), and measures for rumination, dissociation, and intolerance of uncertainty. All participants practiced both of the task conditions with Tetris gameplay running for 2 min and D-Corsi for six rounds. The researcher then left the room so that each participant could view the Trauma Film Paradigm on their own. The VAS were then re-administered along with attention to film ratings. Participants then went about their day as usual and returned to the lab after 4-h.

#### Day 0: Afternoon Session

After returning to the lab, measures were completed for activity involvement during the 4-h gap, intrusive memory frequency, perspective, cause, and ratings for intensity and distress associated with the memories were also obtained. During the lab session, participants were asked, “How intense were the intrusive memories? That is, how strongly did you experience them?” and “How distressed were you by the intrusive memories? That is, how upsetting were they for you?”

With the researcher in the room, a film reminder cue was viewed, after which participants were randomly allocated to one of the three experimental conditions: Tetris, D-Corsi, or Control [silence], for 12-min. Next, VAS were administered for intrusive memory frequency and task enjoyment, and participants registered for the Metricwire diary phone app. Instructions were provided for the phone app and study overall, and participants completed a checklist to confirm their understanding of task requirements tasks over the next week.

#### Days 0–8

Using the Metricwire app, participants monitored intrusive memory intensity and distress at night on Day 0, and then three times daily on Days 1–7. An email link with the final online questionnaires was sent to participants on Day 8. Participants who completed all components of the study were sent the e-gift voucher and thanked for their participation it the study.

### Statistical Analysis

Between group differences for attention to the film, diary accuracy and number of entries, task enjoyment, self-report measures, VAS for levels of distress and being bothered by the film, re-experiencing, intensity, and distress ratings following the 4-h break were analyzed using one-way ANOVAs with Bonferroni-corrected pairwise comparisons. Data for changes in VAS scales from pre- to post-film viewing for sad, hopeless, fearful, horrified and depressed were analyzed using ANCOVAs with Bonferroni-corrected pairwise comparisons, with the pre-film rating entered as the covariate. Data for intensity and distress scores were collected at 22 time points across Days 0–7, and were analyzed using Linear Mixed Models (LMM) with Bonferroni-correct pairwise comparisons. A first order autoregressive covariance structure was specified. Timepoint, condition and the interaction of timepoint by condition were included as fixed effects and a random intercept was specified. Bonferroni adjustments were used to control the type 1 error rate for pairwise comparisons. Prior to running the LMM, correlational analyses between the key variables of total intrusive memory frequency, and average intensity and distress scores were conducted using data from Days 0–7. Outcomes are reported in Supplementary Table 1 for the interested reader.

## Results

Data for 107 participants aged 18–68 were analyzed. The group sizes were Control, *n* = 36; Tetris, *n* = 35; D-Corsi, *n* = 36. Data for demographic characteristics, task and compliance ratings, self-report questionnaires, and pre- and post-Trauma Film Paradigm measures are reported in Table 1. Of note is that intrusion intensity and distress were correlated 0.93. We nonetheless report each of these ratings separately for consistency with our pre-registration and on the basis that creating a combined composite score for these two items would have resulted in a variable with an unclear conceptual definition.

**Table 1:** Sample, task and compliance measures, self-report measures.

**Sample characteristics**	**Condition**							**Between conditions comparisons**
		**Control (*****n*** **= 36)**		**Tetris (*****n*** **= 35)**		**D-Corsi (*****n*** **= 36)**		
		**Mean**	**SD**	**Mean**	**SD**	**Mean**	**SD**	
**Age (years)**		27.36	10.64	29.06	9.77	30.43	11.13	*F*_(2, 104)_ = 0.49
		***n***	**%**	***n***	**%**	***n***	**%**	
**Gender**								
	Female	25	23.3	23	21.5	19	17.7	
	Male	11	10.2	12	11.2	17	15.8	
**First language**								
	English	17	47.2	23	65.7	23	63.9	
	Asian	16	44.4	9	25.7	12	33.3	
	Other	3	8.3	3	8.6	1	2.8	
Main occupation								
	Student	29	80.6	24	68.6	21	58.3	
	Other	7	19.4	11	31.4	15	41.7	
**Task and compliance measures (Scale: 0–100)**								
		**Mean**	**SD**	**Mean**	**SD**	**Mean**	**SD**	
Attention to film		97.28	4.68	93.94	8.41	95.75	6.34	*F*_(2, 104)_ = 2.25
Distress for the film		74.06	26.36	77.40	20.95	72.06	24.27	*F*_(2, 104)_ = 0.45
Bothered by the film		69.69	21.20	71.74	19.22	69.36	25.15	*F*_(2, 104)_ = 0.89
Diary accuracy		88.94	10.95	89.69	12.84	88.71	12.76	*F*_(2, 104)_ = 0.06
Task enjoyment***		48.67	27.15	73.11	25.53	61.33	26.78	*F*_(2, 104)_ = 7.56
		**Pairwise comparisons**		**Mean difference**		**SE**		
		Control, Tetris^***^		−24.45		6.29		
		Control, D-Corsi		−12.67		6.25		
		Tetris, D-Corsi		11.78		6.29		
		***n***	**SD**	***n***	**SD**	***n***	**SD**	
Diary entries (maximum = 22)		19.81	2.58	20.09	2.48	19.92	2.62	*F*_(2, 104)_ = 0.11
		**Mean**	**SD**	**Mean**	**SD**	**Mean**	**SD**	
**Self-report measures**								
	Depression	13.53	4.62	12.23	3.34	12.42	3.41	*F*_(2, 104)_ = 0.31
	Anxiety	16.69	5.20	16.94	5.81	16.36	4.89	*F*_(2, 104)_ = 0.90
	IES-R	15.03	5.62	14.31	4.63	14.50	5.06	*F*_(2, 104)_ = 0.83
		**Mean**	**SE**	**Mean**	**SE**	**Mean**	**SE**	
**Estimated marginal means post-film**								
	Sad	54.99	4.43	54.03	4.50	47.32	4.43	*F*_(2, 103)_ = 0.089
	Hopeless	33.48	4.47	27.08	4.50	26.88	4.45	*F*_(2, 103)_ = 0.70
	Fearful	46.70	5.11	37.44	5.19	41.82	5.10	*F*_(2, 103)_ = 0.80
	Horrified	51.61	5.24	52.29	5.31	52.30	5.22	*F*_(2, 103)_ = 0.01
	Depressed	39.56	4.20	34.39	4.26	28.70	4.19	*F*_(2, 103)_ = 1.67
**Ratings post-4 h break**								
		**Mean**	**SD**	**Mean**	**SD**	**Mean**	**SD**	
	Intensity	52.00	27.72	42.82	21.85	44.18	26.58	*F*_(2, 100)_ = 1.31
	Distress	41.18	24.68	40.82	27.99	47.67	28.95	*F*_(2, 100)_ = 0.70

*n = 107*.

*** *p < 0.001*.

*IES-R, Impact of Events Scale-Revised*.

### Pre-task Group Equivalency of Analog Trauma Induction

Group equivalency analyses were undertaken prior to hypothesis testing. Increased negative emotional valence was reported across all conditions from pre- to post-Trauma Film Paradigm. No between-group differences were indicated for attention to the film, diary accuracy or number of entries, self-report measures, VAS scores for distress or being bothered by the film, or VAS score changes for emotionality associated with viewing the film.

Intensity and distress levels for participants who reported the presence of intrusive memories for the Trauma Film Paradigm during the 4-h break did not differ between groups. Outcomes for rumination, dissociation, and intolerance of uncertainty are beyond the scope of this paper and will be reported elsewhere.

### Post-task Analyses

#### Task Enjoyment

Participants in the Tetris condition reported greater task enjoyment than those in the Control group but no other between-group differences were reported.

#### Analyses of Average Scores Between Conditions

##### Mean Intensity Scores

There were no between-group differences for the mean intensity scores (*p* > 0.05) across Days 1–7. Mean scores for the conditions were: Control = 2.86 (*SE* = 0.25); Tetris = 2.89 (*SE* = 0.26); D-Corsi = 2.74 (*SE* = 0.25).

##### Mean Distress Scores

There were no between-group differences for mean distress scores (*p* > 0.05) across Days 1–7. Mean distress scores for the conditions were: Control = 2.40 (*SE* = 0.26); Tetris = 2.32 (*SE* = 0.27); D-Corsi = 2.18 (*SE* = 0.25).

##### Re-experiencing

There were no between-group differences on the IES-R, *F* (2, 104) = 0.19, *p* = 0.83.

### Primary Analyses

#### Days 0–7

Our central hypothesis pertained to the main effects of the visuospatial task conditions on reducing the perceived intensity and distress of intrusive memories over the course of a week, as compared to the control condition.

For intensity, results indicated a significant main effect for time (*F* = 14.50, *df* = 1, 344.23, *p* = <0.001), but not for condition *(F* = 0.74, *df* = 2, 252.68, *p* > 0.477) or the interaction of condition by time (*F* = 1.01, *df* = 2, 344.25, *p* > 0.366).

For distress, results indicated a significant main effect for time (*F* = 7.14, *df* = 1, 341.05, *p* = <0.008), but not for condition (*F* = 0.77, df = 2, 258.44, *p* > 0.463) or the interaction of condition by time (*F* = 0.74, *df* = 2, 341.05 *p* > 0.480).

## Discussion

Cognitive tasks involving a visuospatial intervention have been shown to influence the trajectory of intrusive memory frequency in analog trauma samples [e.g., (6, 8)]. However, the subjective intensity and distress associated with these memories has not been subject to extensive research. The present paper builds on these previous findings with a focus on these potentially more informative outcomes. Importantly, our analysis is derived from data reported in a previous paper which demonstrated benefit so far as the frequency of intrusions is concerned, suggesting that the study protocol was appropriately administered.

Our hypotheses that participants in either of the visuospatial task conditions (the Tetris intervention or D-Corsi) would report lower levels of intensity and distress for intrusive memories as compared to participants in the control condition was not supported. This finding is broadly similar to the results of Meyer et al. (21); at least so far as intrusion-related distress is concerned. However, our results differ from studies involving eye-movement only tasks wherein clinical (20) and non-clinical (18, 19) participants reported lower levels of intensity for negatively valenced autobiographical memories. In our study, the Trauma Film Paradigm appeared to sufficiently evoke intense and distressing intrusive memories in each group, discounting the possibility that a lack of overall between-group differences in intensity and distress were a product of insufficient or unequal levels of initial activation.

Instead, it remains possible that differences in the intervention between our study and that of others explain the discrepancy in results. The visuospatial tasks of the present study presumably tax working memory to a greater extent than the eye movement only tasks of Lilley et al. (20) and others. Thus, research suggesting that the taxing of cognitive resources during encoding is important (12) may only apply so far as the frequency of intrusive memories are concerned. Further research should aim to confirm the findings from previous eye movement tasks with the aim of identifying the unique features of these tasks that confer benefit so far as intensity and distress are concerned.

## Limitations

Limitations of the current study include the use of a single-item measure for intensity and upsetting, rendering each variable's reliability indeterminate. However, the need for brevity given that these measures were administered at three time points daily, as well as the absence of validated assessment tools for these constructs, resulted in our reliance on single item assessments, much as in previous studies (19, 20). A further limitation was the use of a Trauma Film Paradigm rather real-life trauma memories. Future research may seek to use idiosyncratic trauma memories, where possible, given that autobiographical trauma memories would likely contain personally relevant and aversive information as compared to a Trauma Film Paradigm. However, in the current study, the use of the Trauma Film Paradigm allowed for consistency of the analog trauma event across the three conditions, which is important for ensuring that any between group differences are not an artifact of group-level differences in the characteristics of people's trauma. A further limitation is that we would have ideally in some way measured participants' pre-experimental levels of cognitive control, given that deficits of cognitive control have been associated with PTSD (32). We nonetheless note that the random allocation of participants to condition may have helped to ensure that there were not any systematic differences in cognitive control between groups. Finally, our study focused on memory consolidation processes rather than re-consolidation of memory following a delay. Whether the present findings extend to memory reconsolidation remains an open question and should be a focus for further research.

## Conclusion

Intrusive memories are a core feature of PTSD and have been associated with overall symptom severity (4, 33). The current study investigated the influence of two visuospatial cognitive tasks, a Tetris intervention and pattern tapping, on emotionality for intrusive memories over the course of a week for participants who were administered the trauma film paradigm. Superiority for reducing levels of intensity or distress for the film was not indicated for either visuospatial task condition, when compared to the control condition. There may be merit in further investigation of these tasks with people who have directly experienced real-life traumatic events so as to ascertain whether, from a clinical perspective, these tasks can influence not only intrusive memory frequency, but also intensity and distress. This will also help to inform clinical translation in relation to integrating mechanistic tasks with psychotherapy processes in order to be best support trauma-exposed individuals.

## Data Availability Statement

The datasets presented in this study can be found in online repositories. The names of the repository/repositories and accession number(s) can be found below: Study protocols, materials and de-identified data are accessible at https://doi.org/10.17605/OSF.IO/VUTCW.

## Ethics Statement

Ethical approval was obtained from the Human Research Ethics Committee at the University of Technology Sydney (ETH17-1455). The patients/participants provided their written informed consent to participate in this study.

## Author Contributions

AB developed the study design under the supervision of, and in consultation with DB. Testing and data collection were performed by AB. AB and DB contributed to the development of the study concept, performed data analysis and interpretation, and drafted the paper, and ZS provided critical revisions. All authors approved the final version of the paper for submission.

## Funding

This research was supported by an Australian Government Research Training Program Scholarship awarded to AB and by a National Health and Medical Research Council (NHMRC) Early Career Fellowship (GNT1122203) awarded to DB. The NHMRC had no role in the design of the study, data collection, data analysis, interpretation of data, or in the writing or revision of the manuscript.

## Conflict of Interest

The authors declare that the research was conducted in the absence of any commercial or financial relationships that could be construed as a potential conflict of interest.

## Publisher's Note

All claims expressed in this article are solely those of the authors and do not necessarily represent those of their affiliated organizations, or those of the publisher, the editors and the reviewers. Any product that may be evaluated in this article, or claim that may be made by its manufacturer, is not guaranteed or endorsed by the publisher.
