# Supplementary material for: Visuospatial Working Memory Tasks May Not Reduce the Intensity or Distress of Intrusive Memories
Source: Front Psychiatry. 2022 Feb 3;13:769957. doi: 10.3389/fpsyt.2022.769957 (PMC8851055; doi:10.3389/fpsyt.2022.769957)
Supplement: Supplementary file 1 [file Table_1.DOCX]

**Supplementary Table 1**

Correlations: Total intrusive memory frequency, and average intensity and distress scores

| \|  \| Intrusive Memory Frequency  (Total Days 0 to 7) \| Intensity  (Average Days 0 to 7) \| Upsetting  (Average Days 0 to 7) \| \| --- \| --- \| --- \| --- \| \| Intrusive Memory Frequency  (Total Days 0 to 7) \| 1 \|  \|  \| \| Intensity  (Average Days 0 to 7) \| .59^***^ \| 1 \|  \| \| Upsetting  (Average Days 0 to 7) \| .59^***^ \| .93^***^ \| 1 \|   *N*  = 107. **** p* < .001  *Note.* Correlations calculated using Pearson’s bivariate correlation coefficient (*r*). |
| --- | --- | --- | --- | --- | --- | --- | --- | --- | --- | --- | --- | --- | --- | --- | --- | --- |
